# Basement membrane-like structures containing NTH α1(IV) are formed around the endothelial cell network in a novel in vitro angiogenesis model

**DOI:** 10.1152/ajpcell.00353.2018

**Published:** 2019-06-12

**Authors:** Yongchol Shin, Akane Moriya, Yuta Tohnishi, Takafumi Watanabe, Yasutada Imamura

**Affiliations:** ^1^Department of Chemistry and Life Science, Kogakuin University, Hachioji, Japan; ^2^Graduate School of Engineering, Kogakuin University, Hachioji, Japan; ^3^Department of Veterinary Medicine, Rakuno Gakuen University, Ebetsu, Japan

**Keywords:** angiogenesis, ascorbic acid, basement membrane, coculture spheroid, nontriple helical polypeptide of type IV collagen α1 chain

## Abstract

Angiogenesis is a process through which new blood vessels are formed by sprouting and elongating from existing blood vessels. Several methods have been used to replicate angiogenesis in vitro, including culturing vascular endothelial cells on Matrigel and coculturing with endothelial cells and fibroblasts. However, the angiogenesis elongation process has not been completely clarified in these models. We therefore propose a new in vitro model of angiogenesis, suitable for observing vascular elongation, by seeding a spheroid cocultured from endothelial cells and fibroblasts into a culture dish. In this model, endothelial cells formed tubular networks elongated from the spheroid with a lumen structure and were connected with tight junctions. A basement membrane (BM)-like structure was observed around the tubular network, similarly to blood vessels in vivo. These results suggested that blood vessel-like structure could be reconstituted in our model. Laminin and type IV collagen, main BM components, were highly localized around the network, along with nontriple helical form of type IV collagen α1-chain [NTH α1(IV)]. In an ascorbic acid-depleted condition, laminin and NTH α1(IV) were observed around the network but not the triple-helical form of type IV collagen and the network was unstable. These results suggest that laminin and NTH α1(IV) are involved in the formation of tubular network and type IV collagen is necessary to stabilize the network.

## INTRODUCTION

Angiogenesis is a process of new blood vessel formation from preexisting vasculature. It plays important roles in several physiological or pathophysiological processes such as wound healing, female reproductive cycles, and tumor growth and metastasis (6). In the process, endothelial cells (ECs) form a cylindrical tubular structure with a central lumen where the blood circulates (7, 13, 31, 39, 40). During lumen formation, plasma membrane proteins or cellular proteins of ECs are distributed in different patterns at the luminal (apical) and basal side to exert endothelial cell polarization.. Basement membranes (BM) are uniformly thin and continuous sheets, formed at the basal sides of epithelial cells, such as ECs and mesothelial cells, and around muscle, fat, and Schwann cells (16, 22, 32, 33, 46). The BM not only helps maintain tissue structure but also the filtration barrier, determination, and maintenance of cell polarity, metabolism, cell viability and proliferation, induction of differentiation, and formation of cell migration pathways. BMs consist mainly of laminin, type IV collagen, nidogen, and heparan sulfate proteoglycans such as perlecan. Type IV collagen is critical for the proper formation of the BM (16). Lack of ascorbic acid (AA) in the diet may lead to scurvy, in which the BM cannot form properly and blood vessels become brittle (29, 44). Since AA promotes proline hydroxylation in collagen, lower hydroxylation levels result in failed triple-helix formation (26). Type I collagen polypeptides that fail to form triple helices are thought to be broken down by the cellular quality control system (35, 37, 43). However, the nontriple helical type IV collagen α1 [NTH α1(IV)] chain is secreted from human fetal lung fibroblasts (TIG-1), mesangial cells, or ECs in the absence of AA (42, 45). Endodermal cells that originate from teratocarcinomas also secrete NTH α1(IV) (10). NTH α1(IV) has been detected in human placenta, using specific antibodies (15) and in rabbit BMs from endomysium of skeletal muscle tissue, nerve tissue, Bowman’s capsule, and renal tubule in the kidney (41). During angiogenesis, BM is constructed around elongated ECs (16). A rabbit angiogenic model in corneal tissue has shown colocalization of triple-helical type IV collagen and NTH α1(IV) around the EC network. In the neovascular tip region of ECs, NTH α1(IV) has been exclusively observed; however, triple- helical type IV collagen has not been detected (41). NTH α1(IV) may play a physiological role in angiogenesis.

Some in vitro angiogenesis models use ECs cultured on collagen gels (18), mixed gels with extracellular matrix (ECM) components (1), or Matrigel (11), whereas coculturing ECs with cells that produce ECM proteins can form an EC network without using an exogenous matrix (5, 9, 21). A 2D (monolayer) or 3D (spheroid) coculture of ECs (19) with fibroblasts reproduces the EC network formation. In this study, we propose a novel angiogenesis model, the 2.5D coculture system, in which cocultured spheroids (3D) spread horizontally on plane substrates (2D), such as culture dishes or cover glasses. In the system, we easily observe the sprouting and elongating processes of EC tubular network from the spheroids adhered onto the substrate. By using this new method, we can study BM formation around the EC tubular network with regard to localization of ECM components, including NTH α1(IV), and the effect of AA on production of type IV collagen. We also discuss the possible involvement of NTH α1(IV) in BM formation and network stability.

## MATERIALS AND METHODS

### 

#### Cell culture.

Human umbilical vein endothelial cells (HUVECs) from Lonza (Walkersville, MD) were used from passages 4 to 9 and cultured in EBM-2 supplemented with growth factors and fetal bovine serum (FBS), as provided in the EGM-2 BulletKit (Lonza). In the case of AA-depleted culture, we depleted AA from EBM-2. Human diploid fetal lung fibroblasts (TIG-1) from the Japanese Collection of Research Bioresources Cell Bank were grown in Dulbecco’s modified Eagle’s medium (Sigma-Aldrich Japan, Tokyo, Japan) containing 10% FBS. Cultures were incubated in a humidified atmosphere with 5% CO_2_ at 37°C.

#### Coculture spheroid.

Coculture spheroids consisting of HUVEC and TIG-1 cells were grown in EBM-2 on 1.5% agarose-coated 96-well plates or in methyl cellulose contained EBM-2 on low-adherent 96-well-rounded bottom plates. Cocultured spheroids from either agarose-coated plates or low-adherent plates were harvested and cultured on plastic dishes or cover glasses. The cover glasses were coated with 30 µg/mL of fibronectin (FN) to 4°C overnight and then washed with phosphate-buffered saline (PBS; 0.8% NaCl, 0.02% KCl, 0.115% Na_2_HPO_4_, and 0.02% KH_2_PO_4_).

#### Immunofluorescence.

Cells were washed by PBS and fixed by 4% paraformaldehyde for 10 min. After fixation, cells were treated with 0.2% TritonX-100 in PBS for 2 min, following by washing with 0.1% Tween20 contained PBS (T-PBS), three times. Cells were incubated with primary antibodies at room temperature and then were washed in T-PBS and incubated with second antibodies. Final washes were performed with T-PBS. Cells were then examined by fluorescence microscopy. HUVECs were labeled with antibodies against human platelet endothelial cell adhesion molecule 1/CD31 (89C2; Cell Signaling) or von Willebrand factor (vFW) (ab6994; Abcam). ECM proteins were labeled with following antibodies: type I collagen (F-56; Daiichi Fine Chemical), type VI collagen α1 chain (AP6587A; Abgent), laminin (L9393; Sigma-Aldrich), and FN (ab26245; Abcam). Antibodies against type IV collagen (JK199) (16) and NTH α1(IV) (JK132) (14, 42) have been previously described. Secondary antibodies were as follows: Alexa Fluor 594 goat anti-mouse IgG (A-11032; Thermo Fisher) and Alexa Fluor 488 goat anti-rabbit IgG (A-11034; Thermo Fisher). Nuclei were stained by DAPI (D1306; Thermo Fisher). Phase-contrast images and fluorescence images were acquired by fluorescence microscopy (FSX100; Olympus). Z-stack images were obtained using fluorescence microscopy (BZ-X700; Keyence) and confocal laser microscopy (Olympus; FV1200). The fluorescence signal and the projected area of the spheroids were quantified using ImageJ software. Angiogenic parameters, such as average vessel length, branching index, or end points, of the EC network were measured using AngioTool software (48). Statistical significance was analyzed by one-way ANOVA with a Tukey’s post hoc test (https://astatsa.com/OneWay_Anova_with_TukeyHSD/).

#### Electron microscopy.

Cells on cover glasses were fixed with modified Karnovsky’s fixative solution (2% paraformaldehyde and 2.5% glutaraldehyde in 0.1 M cacodylate buffer) overnight at 4°C, followed by washing with 0.1 M cacodylate buffer for 5 min, three times. Cells were postfixed with 1% osmium tetroxide in 0.1 M cacodylate buffer for 30 min, dehydrated through an ethanol gradient, transferred to QY-1 (Nisshin EM, Tokyo, Japan), and embedded in epoxy resin (Quetol 812, Nisshin EM) according to standard procedures. Ultrathin sections of the network around the adhered spheroid were prepared using an ultramicrotome (Supernova; Reichert-Jung, Vienna, Austria) and mounted on copper grids. Ultrathin sections were examined under a transmission electron microscope (TEM; JEM 1400; JEOL, Tokyo, Japan) at an acceleration voltage of 80 kV.

## RESULTS

### 

#### Formation of cocultured spheroids and HUVEC network formation.

**Fig. 1.**
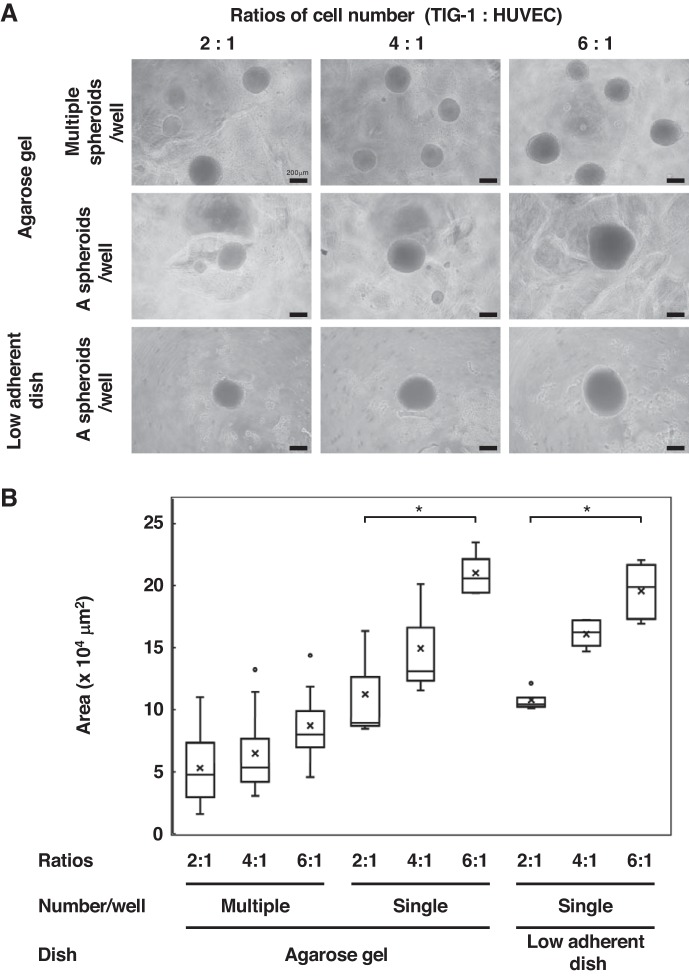
Formation of cocultured spheroids on low-adherent culture dishes or agarose gel. Fibroblasts (TIG-1) and human umbilical vein endothelial cells (HUVECs) were mixed in different ratios (2:1, 4:1, or 6:1) and seeded on low-adherent culture dishes in medium containing 0.25% methylcellulose or on agarose gel with 4 × 10^4^ HUVECs/well. *A*: phase-contrast images of coculture spheroids with HUVECs and different number of TIG-1 are shown. *B*: the size of projected area of the spheroid on each image was obtained using ImageJ software. The data are presented as box plot with whiskers; median values are shown by the line that divides the box into two parts; mean values are represented with an “x.” Differences among three experimental groups using different cell ratios such as 2:1, 4:1, or 6:1 were analyzed by one-way ANOVA with post hoc Tukey honestly significant difference. Results represent at least 3 experiments. *P < 0.01.

We seeded TIG-1 and HUVEC cells at different ratios (2:1, 4:1, or 6:1) with a constant number of 4 × 10^3^ HUVEC/well. After 7 days, cocultured spheroids had formed on either agarose gels or low-adherent plates (Fig. 1*A*). On the low-adherent dishes in a medium that contained 0.25% methylcellulose, only one spheroid was formed in a well. However, on the agarose gel, multiple spheroids of different sizes were often formed in a single well. The size of projected area of the spheroid on each image was obtained using ImageJ software (Fig. 1*B*). When the number of TIG-1 cells was increased, the size of spheroids increased in the low-adherent dish, whereas both volume and number of spheroids increased in wells on the agarose gel. We considered the round bottom shape of the low-adherent dish to be preferable in accumulating cells, followed by the formation of a single spheroid in a well. As spheroids of similar sizes were consistently obtained, the cocultured spheroids formed in low-adherent plates were used for further experiments.

**Fig. 2.**
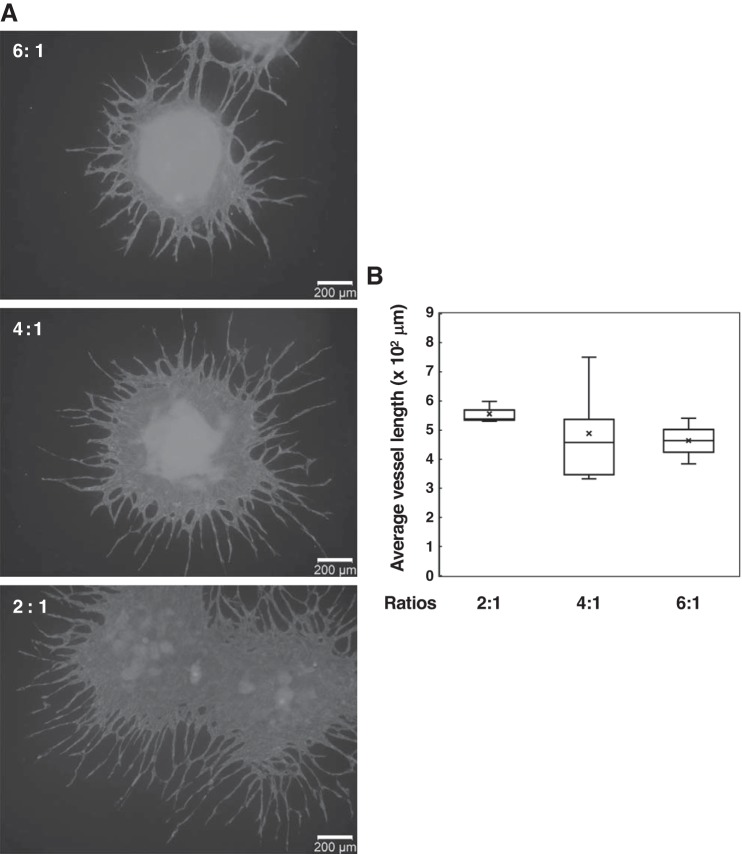
Analysis of sprouting and elongation of endothelial cells (ECs) from cocultured spheroids of human umbilical vein endothelial cells (HUVECs) with different ratios (6:1, 4:1, or 2:1) of fibroblasts (TIG-1) and HUVEC cells, seeded in medium containing 0.25% methylcellulose on low-adherent culture dishes with 4 × 10^4^ HUVECs/well. Cocultured HUVECs and TIG-1 formed spheroids, which were transferred to 4- or 24-well culture dishes and cultured for 7 days. *A*: gray: EC networks (anti-CD31 antibody, Alexa Fluor 594 goat anti-mouse IgG). *B*: the average vessel length of HUVEC network from the spheroid on each image was quantified using AngioTool software. The data are presented as box plot with whiskers as shown in Fig. 1*B*. Results represent at least three experiments.

**Fig. 3.**
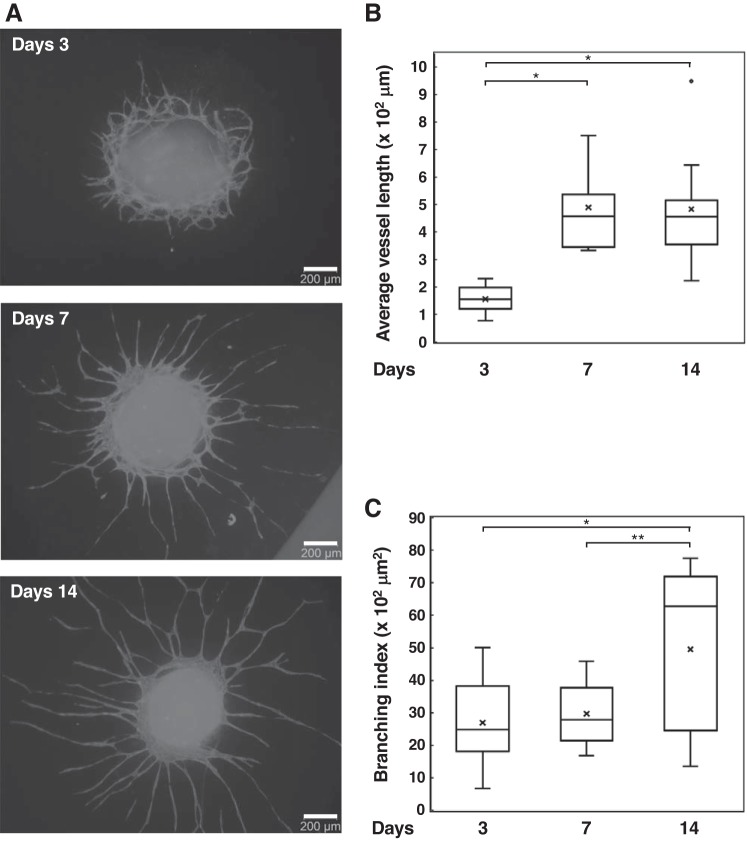
Time-dependent analysis of sprouting and elongation of endothelial cell (EC) network from cocultured spheroids of fibroblasts (TIG-1) with human umbilical vein endothelial cells (HUVECs) at a ratio of 4:1, which were formed in medium containing 0.25% methylcellulose in low-adherent culture dishes with 4 × 10^4^ HUVECs/well. One spheroid was transferred onto a culture dish and cultured for 14 days. *A*: gray: EC networks (anti-CD31 antibody, Alexa Fluor 594 goat anti-mouse IgG). *B* and *C*: the average vessel length (*B*) and branching index (*C*) of HUVEC network from the spheroid on each image were quantified using AngioTool software. The data are presented as box plot with whiskers as shown in Fig. 1*B*. Results represent at least three experiments. *P < 0.01; **P < 0.05.

Spheroids with different TIG-1/HUVEC ratios were seeded onto adherent culture dishes and cultured for 7 days (Fig. 2). Networks consisted of HUVECs extending from spheroids with different ratios of TIG-1 and HUVECs (Fig. 2*A*). Furthermore, we quantified the average vessel length of HUVEC network (Fig. 2*B*). Here, we defined a vessel as a segment between two branching points or a branching point and an end point. There was no significant difference among the average vessel length of HUVEC network from spheroids formed with 8, 16, or 24 × 10^3^ of TIG-1 (designated 2:1, 4:1, or 6:1 in Fig. 2, respectively), whereas spheroids formed from 40 × 10^3^ of TIG-1 and 4 × 10^3^ of HUVEC could not adhere to the plastic dishes (data not shown). TIG-1 cells spread more widely beyond the HUVEC network (data not shown). Figure 3 shows the elongation of HUVEC network from spheroids formed from 16 × 10^3^ of TIG-1 and 4 × 10^3^ of HUVECs as a function of incubation time. At *day 3*, HUVEC networks surrounded the outside of the adhered spheroid, had spread radially from the spheroid at *day 7*, and were more elongated at *day 14* (Fig. 3*A*). Average vessel length at *day 7* was longer than the length at *day 3*; however, it was similar to the length at *day 14* (Fig. 3*B*), whereas the branching index, that is a number of branching points per unit area, at *day 14* increased almost twofold of the branching index at *day 3* or *day 7* (Fig. 3*C*). Those results suggest that in 2.5D coculture system HUVECs transform their morphology to tube-like structures and then the tubular structures are elongated up to around 500 μm, followed by branching to create the vessel-like networks.

#### Localization of ECM proteins around HUVEC network.

**Fig. 4.**
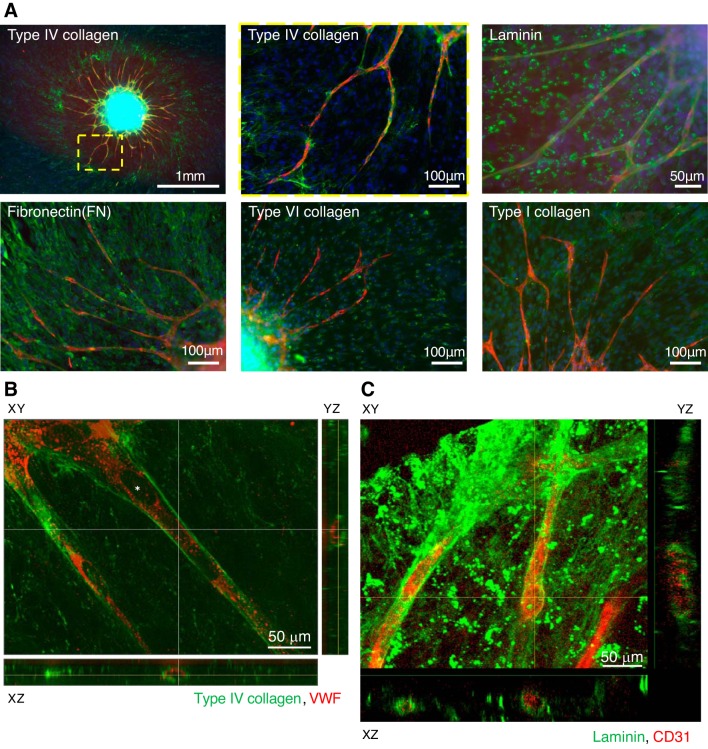
Localization of extracellular matrix (ECM) proteins around endothelial cell (EC) network. *A*: coculture spheroids with fibroblasts (TIG-1) with human umbilical vein endothelial cells (HUVECs; 4:1) were cultured for 7 days with 4 × 10^4^ HUVECs/well. Red: EC networks (anti-CD31 or anti-von Willlebrand factor (vWF) antibody, Alexa Fluor 594-labeled secondary antibodies); green: ECM proteins (specific antibodies, Alexa Fluor 492-labeled secondary antibodies); blue: cell nuclei (DAPI). *B*: coculture spheroids with TIG-1 with HUVECs (4:1) were cultured for 7 days with 4 × 10^4^ HUVECs on the cover glass. Red: EC networks (anti-VWF antibody); green: type IV collagens (JK199 specific antibody). Z-stack images were obtained using fluorescence microscopy (Keyence BZ-X700) and show the space in the network (*). Results represent at least three experiments. *C*: coculture spheroids with TIG-1 with HUVECs (4:1) were cultured for 7 days with 4 × 10^4^ HUVECs on the cover glass. Red: EC networks (anti-CD31 antibody); green: laminin (specific antibody). Z-stack images were obtained using confocal laser microscopy (Olympus; FV1200). Results represent at least three experiments.

Microvascular BM has been widely shown to consist of ECM proteins, such as laminin, type IV collagen, perlecan, nidogen, and so on. To address the localization of BM proteins around the HUVEC network, TIG-1 and HUVEC cells were cocultured in a 2.5D procedure for 7 days and then immunostained with antibodies against ECM proteins (Fig. 4*A*). Type IV collagen and laminin were located along the network. FN was also located along the network but not type I and type VI collagens. In TIG-1 cells, laminin, FN, and type I and type VI collagens were detected, but type IV collagen was very weakly distributed on TIG-1. Nucleus staining with DAPI (blue) showed TIG-1 spread more widely beyond the HUVEC network. To clarify whether the network was surrounded by type IV collagens or laminin, we took images of 3D reconstructed networks with a BZ-X700 (Keyence; Fig. 4*B*) or FV1200 (Olympus; Fig. 4*C*), which showed type IV collagens or laminin (green) surrounding the EC network (red) in a cylindrical shape as a BM, respectively. We could not identify a space in the networks as a nucleus or a lumen. These results suggest that the HUVEC network is surrounded by BM components, laminin, type IV collagen, and FN.

**Fig. 5.**
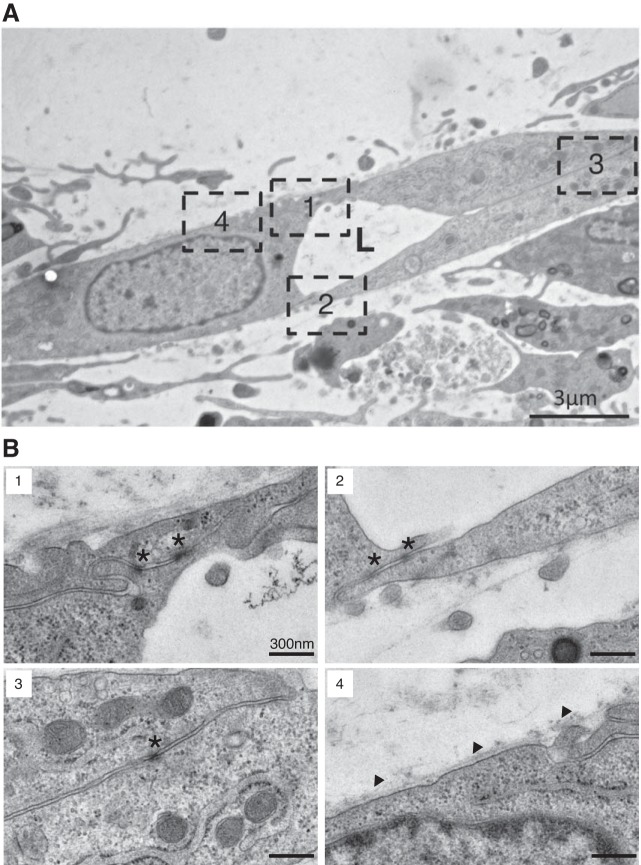
Transmission electron microscope images of elongating endothelial cell (EC) network from cocultured spheroids. *A*: coculture spheroids with fibroblasts (TIG-1) with human umbilical vein endothelial cells (HUVECs) (4:1) were cultured for 6 days with 4 × 10^4^ HUVECs on cover glass coated with fibronectin; ultrathin sections of EC network were electron-stained; spheroids display lumen formation (L) by ECs. Results represent at least three experiments. *B*: higher magnification of areas depicted in *A*. Asterisk: tight junctions connecting adjacent cells; arrowheads: segmental basement membrane formations.

During angiogenesis, EC cells form a tubular structure that fills with blood in the lumen. As immunofluorescence microscope could not clearly show the lumen, we used TEM analysis. Cocultured spheroids were seeded on FN-coated cover glasses. After 6 days of culture, ultrathin sections of the network were analyzed by TEM. HUVECs were connected with tight junctions and formed lumens (Fig. 5). We found BM-like structures along the HUVECs that formed the lumen but not among cells without the lumen. Together with results of immunostaining with anti-ECM antibodies, we concluded that the microvessel-like structure was reproduced in 2.5D culture system.

#### Effect of AA on the HUVEC network.

**Fig. 6.**
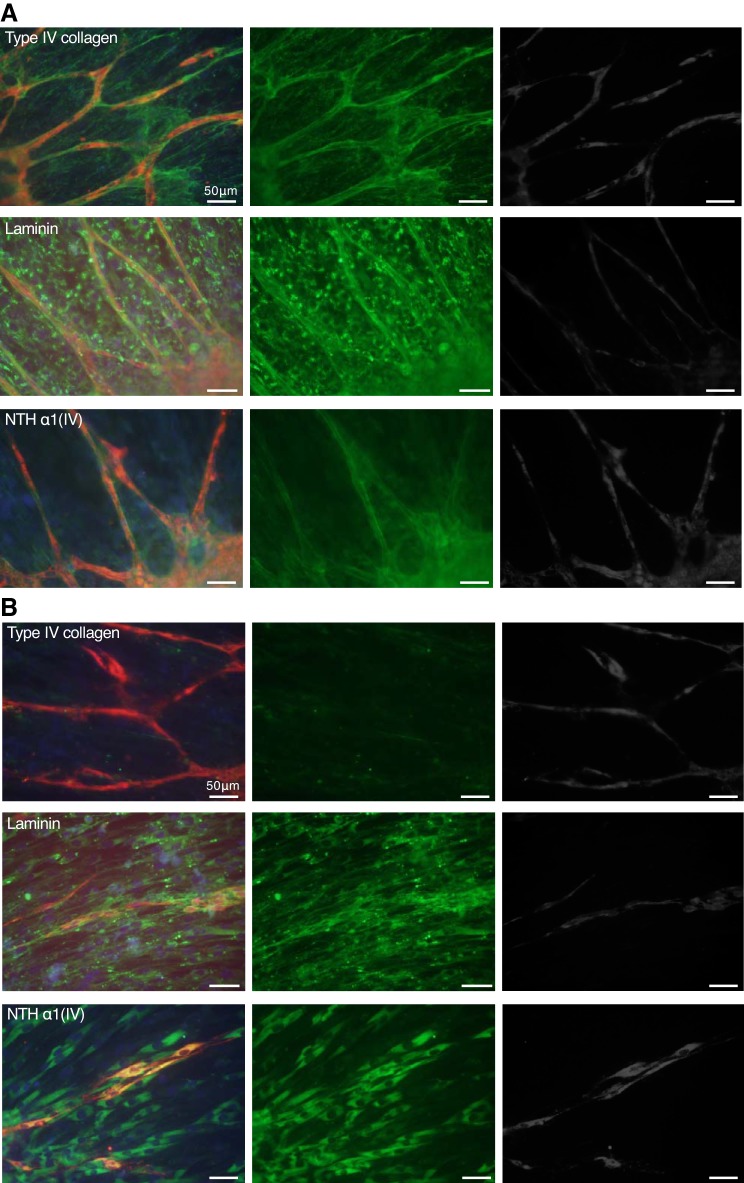
Effect of ascorbic acid (AA) on endothelial cell (EC) network and extracellular matrix (ECM) protein localization. Cocultured spheroids with fibroblasts (TIG-1) with human umbilical vein endothelial cells (HUVECs; 4:1) were cultured for 7 days with 4 × 10^4^ HUVEC/well on the plastic dishes in the medium with (*A*) or without (*B*) AA (2 mM). Red in the merged panel and gray in the separate channel: EC networks (anti-CD31 or anti-VWF antibody, Alexa Fluor 594-labeled secondary antibodies); green: ECM proteins (specific antibodies, Alexa Fluor 492-labeled secondary antibodies); blue: cell nuclei (DAPI). Results represent at least three experiments. NTH α1(IV), nontriple helical polypeptide of type IV collagen α1 chain.

Type IV collagen forms triple-helical structures in the presence of AA. Depletion of AA causes secretion of NTH α1(IV) from several culture cells, including TIG-1. As NTH α1(IV) was thought to mediate dynamics of the vascular system in a rabbit angiogenic model (41), we examined the production and deposition of NTH α1(IV) in the HUVEC networks of coculture spheroids. To identify triple-helical or nontriple helical type IV collagen, we used conformation-specific antibodies. JK-199 reacts only with type IV collagen in the triple-helical conformation (17); JK-132 binds to type IV collagen α1 chain in nontriple helical form but not with triple-helical type IV collagen (42), and JK132 recognizes amino acids 1165–1179 in the type IV collagen α1 chain: an epitope that is hidden in the triple-helix conformation (14). In the presence of AA, type IV collagens and laminins are located along the network (Fig. 6*A*). Type IV collagens also situate into the spaces between networks. Laminins were intensively located in spots on the TIG-1. Although presence of AA tends to produce the triple-helical form of type IV collagen, NTH α1(IV) was clearly located along the network. Even when AA was depleted, the EC networks sprouted and elongated from the cocultured spheroids as well as they did in the presence of AA (Fig. 6*B*). Triple-helical type IV collagens were rarely sited along the network, whereas laminins were located along the network, and also on TIG-1 cells around the network. NTH α1(IV) was intensively located inside of HUVEC and TIG-1 cells around the network. Considering the results, the basement membrane along the network found in the presence of AA might not be able to form in the AA-depleted condition. Therefore, that the distribution of NTH α1(IV) outside cells could not be detected.

**Fig. 7.**
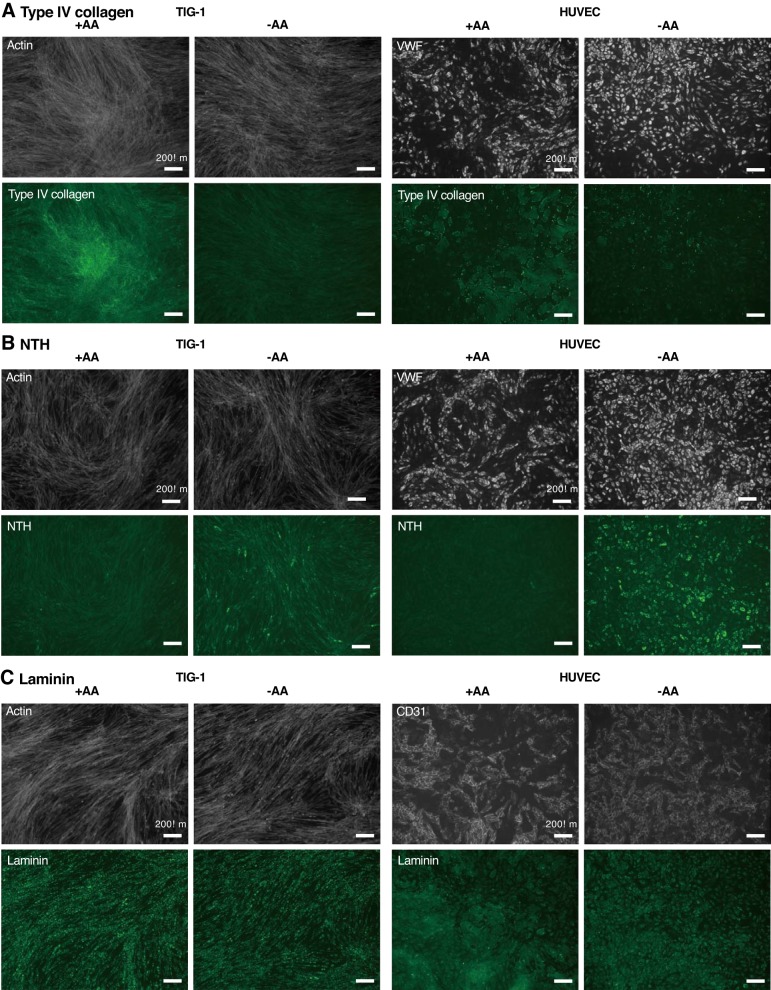
Effect of ascorbic acid (AA) on localization of extracellular matrix (ECM) proteins. fibroblasts (TIG-1) or human umbilical vein endothelial cells (HUVECs) with 10 × 10^4^/well were cultured for 2 or 3 days, respectively, with or without 2 mM AA. Actin fibers in TIG-1 were stained with rhodamine phalloidin (gray). HUVECs were stained with anti-CD31 or anti- von Willlebrand factor (VWF) antibody, followed by Alexa Fluor 594-labeled secondary antibodies (gray). Type IV collagen (*A*), nontriple helical form of type IV collagen α1 chain [NTH α1(IV)] (*B*), or laminin (*C*) was labeled with specific antibodies, followed by Alexa Fluor 492-labeled secondary antibodies (green). Results represent at least three experiments.

Next, we examined expression of ECM proteins from monocultured TIG-1 or HUVEC cells in the presence or absence of AA. In the presence of AA, type IV collagens were deposited in both TIG-1 and HUVEC cells but were only faintly expressed in in the absence of AA in both cell types (Fig. 7*A*). Conversely, NTH α1(IV) was intensively expressed in both cell types without AA but was absent in both cell types with AA (Fig. 7*B*). Laminins were intensively expressed in TIG-1 and HUVECs under both conditions. Especially in TIG-1, laminins were localized to a spot (Fig. 7*C*). These results suggest that triple-helical type IV collagen is not necessary for the formation of HUVEC tubular structures and interaction of HUVECs with TIG-1 induces NTH α1(IV) to localize along the network, even in the presence of AA. Using ImageJ software, we quantified the mean fluorescence intensity of the ECM proteins (green) relative to nucleus (blue) from monocultured TIG-1 or HUVEC cells in the presence or absence of AA. However, we did not find significant differences between the intensities of type IV collagen or NTH α1(IV) proteins in the presence or absence of AA. Cell-based enzyme-linked immunosorbent assay (ELISA) might be useful tool for the quantification of cell-surface proteins (23).

**Fig. 8.**
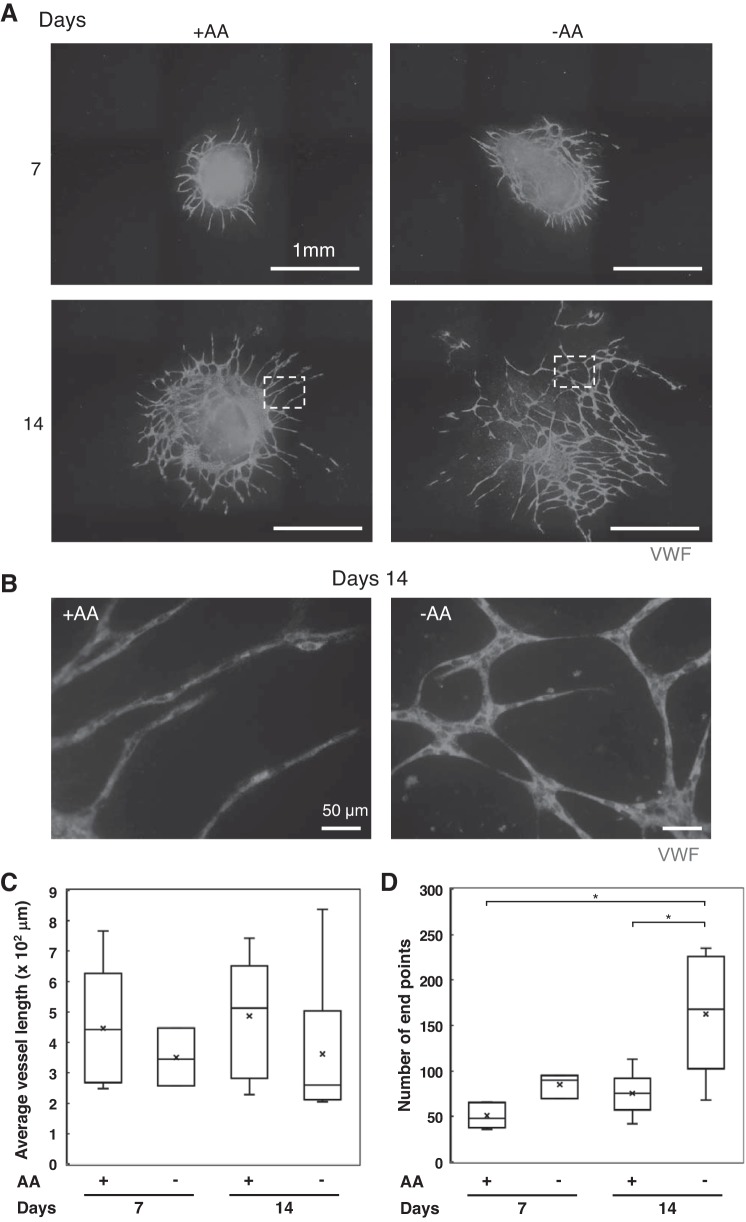
Effect of ascorbic acid (AA) on endothelial cell (EC) network stabilization. Cocultured spheroids with fibroblasts (TIG-1) with human umbilical vein endothelial cells (HUVECs; 4:1) were cultured for 7 or 14 days with 4 × 10^4^ HUVEC/well on the plastic dishes in the medium with or without 2 mM AA. *A*: gray: EC networks [anti-von Willlebrand factor (VWF) antibody, Alexa Fluor 594-labeled secondary antibodies]. *B*: higher magnification of area depicted in *A* shows that network without AA is disconnected and barely elongated. *C* and *D*: the average vessel length (*C*) and number of end points (*D*) of HUVEC network from the spheroid on each image were quantified using AngioTool software. The data are presented as box plot with whiskers as shown in Fig. 1*B*. Results represent at least three experiments. *P < 0.01.

Next, we tested whether the network from cocultured spheroids stabilized in the presence or absence of AA at 7 or 14 days (Fig. 8). At *day 7*, the HUVEC network was similarly formed in both conditions, but at *day 14*, whereas the networks with AA were stable and showed constant tube widths, the network without AA was disconnected (Fig. 8, *A* and *B*). There is no significant difference between average vessel lengths in the absence of AA and in the presence of AA (Fig. 8*C*). However, number of end points of vessel network in the absence of AA increases almost twofold of the number of end points in the presence of AA (Fig. 8*D*). These results suggest that the network formation was formed in either condition until *day 7* but could not be maintained without AA and began to degrade between *day 7* and *day* *14*. Whereas, when we cultured the spheroids with AA for 7 days followed by culturing for 7 days under the AA-depleted condition, the angiogenic parameters, such as average length or end points of the tubular network or the localization of type IV collagen around tubular network from spheroids, were not significantly different from those for 14 days culture with AA. These results suggest that the cultures of the spheroids for 7 days with AA allow the tubular network to stabilize within following 7 days.

## DISCUSSION

In this study, we investigated a novel angiogenesis model, 2.5D coculture system in which HUVEC and TIG-1 formed 3D spheroids and were then seeded onto culture dishes or cover glasses (2.5D), where sprouting and elongating EC tubular networks could be observed. In the model system, interactions between two different type of cells affect the expression and secretion of extracellular matrix proteins, especially NTH α1(IV).

Most common in vitro tubular network-formation assays are culturing HUVECs on Matrigel, BM matrices derived from murine tumors (11), or collagen gels (18). The model systems easily reproduce vessel-like network. However, it is unclear how ECM proteins are deposited into the BM from ECM protein-producing cells, because ECM proteins are exogenously provided in these model systems. To evaluate and analyze the deposition of ECM proteins from the cells and the effect of interaction of different type of cells on the basement membrane matrix assembly, several type of coculture systems have been investigated. Coculture of ECs and fibroblasts on the culture dishes forms vessel-like tubular networks (5). In addition, coculture of ECs and pericytes, which provide ECM proteins, on type I collagen gel has shown that the both cells contribute the deposition of ECM proteins and facilitate the vessel maturation including vascular basement membrane matrix assembly (38). Furthermore, the spheroids formed with HUVECs and fibroblasts on the agarose gels develop the tubular networks inside of the spheroids (19). However, these models cannot assess the sprouting and elongation of the tubular network. Although Heiss et al. (12) have shown the tubular networks are sprouting and elongating from the spheroid formed only with HUVECs on the Matrigel, the model cannot evaluate the deposition of ECM proteins into the BM from ECM protein-producing cells. In our preliminary experiments with a 2D monolayer coculture system, we had noticed cellular aggregates in several parts of the culture dish from which EC tubules spread out (unpublished data). We then tested whether tubular networks elongated from the 3D cocultured spheroids seeded on the culture dishes. The aggregates adhered to culture dishes or FN-coated cover glasses, followed by dispersing fibroblasts ahead of elongation of EC networks. The EC networks displayed lumen formation and tight junctions among ECs. As the elongation of EC network in this model is similar to this process in the aorta ring model (27), we concluded that a 2.5D coculture system could reproduce the elongation of blood vessels in the angiogenesis. Furthermore, in the coculture system, expression and secretion of extracellular matrix proteins were investigated comparing in the monoculture system. In the 2D or 2.5D coculture systems, cell numbers and ratios of the two cell types were crucial for fully elongated network formation. In the 2D model, fibroblast cells had spread over the well surface before the networks began to be clearly recognizable. In the 2.5D model, fibroblasts migrated out from the spheroid before EC elongation and network appeared. Cell numbers and ratios probably affect the sequence of events; thus the fibroblast spread first and then the ECs elongated. These results may be clues to their underlying molecular mechanisms.

Beneath the ECs, microvascular capillaries have BMs, constructed of laminin, type IV collagen, pearlcan, decorin, and so on (16). In our new model, the BM-like structure was formed just beneath the ECs, which were connected with tight junctions and formed the lumen. As the ECs were surrounded by laminin, type IV collagen, and FN, those ECM proteins likely formed BM structures similar to those in capillaries. Coculture of pericytes with EC has led to specific induction of laminin, nidogen, perlecan, and FN and facilitated vessel maturation events (38). Without pericytes, the BM matrix was not observed. Together with our results, we suggest that ECM-producing cells could control BM formation and blood vessel stabilization. Although what induces tubular structure formation in the coculture system is unclear, interaction of the two cell types may affect this process.

NTH α1(IV) is found in human placenta (15) and BMs of several tissues in rabbits (41). In the absence of AA, several cell lines, including TIG-1, secrete NTH α1(IV). With the 2.5D culture system, we detected NTH α1(IV) in the presence of AA but only around the HUVEC networks, although triple-helical type IV collagen was produced by TIG-1 cells independently of HUVECs. These results suggest that interaction between TIG-1 and HUVEC promotes the production and secretion of NTH α1(IV). The tubular EC network could be formed under AA-depleted conditions where laminin and NTH α1(IV), but not type IV collagen, were located around the network as main BM components. Prolonged cultivation without AA caused the EC network to destabilize. Scurvy is a disease caused by a lack of dietary AA (44), which is required as a cofactor for prolyl hydroxylase and lysyl hydroxylase that hydroxylate the proline and lysine residues (respectively) in collagen. These modifications are required to form triple-helical collagen (30, 37, 43). Together with results of ECM localization and the stabilization of network in the presence or absence of AA, these findings indicate that laminin and NTH α1(IV) are rather involved in formation or elongation of the tubular EC network, whereas type IV collagen in triple-helical form is necessary for stable EC network formation. Recently, AA was found to be involved in not only hydroxylation of proline residues in collagen but also demethylation of 5-methylcytosine in DNA, inducing global epigenetic reprogramming. In AA-depleted conditions, EC-EC junctions might be disrupted by reduction of junctional proteins in EC. The effect of AA on gene regulation during tube formation in addition to ECM production needs to be clarified. Laminin is the only BM component needed for BM formation during the peri-implantation period of mammalian blastocyst development of mice (8, 25, 28, 33, 36). Studies using embryonic stem cells and embryoid bodies have shown that laminin polymers form on the surface of the cells with β1 integrin and dystroglycan (2, 20, 34). In the absence of laminin polymer, type IV collagen does not assemble properly into BM-like structures (47). Together with our results, we suggest that plasma membrane proteins such as integrins facilitate the formation of primitive BM-like structures consisting of laminin with NTH α1(IV) and then type IV collagen polymers that associate with the primitive BM on the cell surface to form stable BM. Laminin binds to type IV collagen via nidogen (3) or perlecan (4). The binding of NTH α1(IV) directly to laminin, nidogen, or perlecan should be further investigated. In the rabbit angiogenic model, NTH α1(IV) is located around the entire network including the tip of the blood vessel, whereas type IV collagen is deposited around the network except the tip of the vessel (41). NTH α1(IV) could have a role in angiogenesis, especially in initiating vessel elongation. Abnormal ECM, such as fragmented collagen, can be a stimulator or an inhibitor of angiogenesis (24). NTH α1(IV) might affect sprouting and elongation of EC tubules similarly to the collagen fragments. NTH α1(IV) was intensively located on ECs that formed the network and on TIG-1 cells around the network in our model, similar to the rabbit angiogenesis model in which NTH α1(IV) was located around the entire network including the blood vessel tip. The similarity of NTH α1(IV) expression suggests that angiogenesis in vivo can be mirrored by our 2.5D coculture spheroids. Since HUVECs could form a tubular network in Matrigel (11), the cell-cell interactions between HUVEC and TIG-1 might form an ECM structure similar to Matrigel with NTH α1(IV) along the HUVEC network. Type IV collagen was deposited around the network except the tip of the vessel in the rabbit angiogenesis model but was localized around the entire network in our model. In the rabbit angiogenesis model, blood vessels were formed in the peripheral ocular surface of cornea. Compared with our new model, ECM proteins were already deposited to the cornea, apparently with fewer fibroblasts than that in our new model.

In conclusion, we have developed a novel 2.5D angiogenesis model that has provided evidence that laminin and NTH α1(IV) affect sprouting and elongation of the EC tubular network and triple-helical type IV collagen stabilizes the EC tubular network.

## GRANTS

This work was supported by the Japan Society for the Promotion of Science KAKENHI Grant JP21500432 and in part by a grant from the Strategic Research Foundation Grant-Aided Project for Private Universities from the Ministry of Education, Culture, Sports, Science, and Technology (MEXT) Grant 2014-2018 (S1411005).

## DISCLOSURES

No conflicts of interest, financial or otherwise, are declared by the authors.

## AUTHOR CONTRIBUTIONS

Y.S. conceived and designed research; A.M., Y.T., and T.W. performed experiments; Y.S., A.M., T.W., and Y.I. analyzed data; Y.S., A.M., and Y.I. interpreted results of experiments; Y.S., A.M., and T.W. prepared figures; Y.S. and Y.I. drafted manuscript; Y.S. and Y.I. edited and revised manuscript; Y.S. and Y.I. approved final version of manuscript.
